# The Prevalence and Factors Associated with Workforce Attrition and Intention‐to‐Leave Among Healthcare Workers in New Zealand: A Systematic Literature Review and Meta‐Analysis

**DOI:** 10.1002/snz2.70025

**Published:** 2026-02-18

**Authors:** Mia Clarke, B. Stephen, M. Frecklington, I. Zeng, M. R. Carroll, R. J. Siegert, S. Stewart

**Affiliations:** ^1^ Department of Podiatry Auckland University of Technology Auckland New Zealand; ^2^ Biostatistics Department of Clinical Sciences Management Auckland University of Technology Auckland New Zealand; ^3^ Department of Psychology and Neuroscience Auckland University of Technology Auckland New Zealand

**Keywords:** attrition, healthcare workforce, intention‐to‐leave, meta‐analysis, New Zealand, retention, systematic review, turnover intention

## Abstract

Despite increasing concern about the stability of New Zealand's (NZ) health workforce, no prior synthesis has estimated the prevalence of attrition or intention‐to‐leave. This systematic review and meta‐analysis included 32 studies and followed Preferred Reporting Items for Systematic Reviews and Meta‐Analysis and Meta‐Analysis of Observational Studies in Epidemiology checklist guidelines to estimate these rates and identify associated factors. Attrition was highest among midwives (26.0%, 95% CI: 17.0%−36.0%), doctors (26.0%, 95% CI: 24.0%−27.0%), and those without postgraduate qualifications (29.0%, 95% CI: 26.0%−32.0%). Intention‐to‐leave was most prevalent among midwives (54.0%, 95% CI: 42.0%−66.0%) and in studies conducted between 2000 and 2010 (33.0%, 95% CI: 20.0%−47.0%). These findings highlight substantial workforce instability, with clear variation by profession, time period, and educational level, underscoring the need for targeted retention strategies to support the sustainability of NZ's healthcare system.

## 1 Introduction

The global healthcare workforce is under increasing pressure, with both current and projected shortages posing substantial threats to the continuity, quality, and equity of healthcare delivery. The World Health Organisation forecasts a global shortfall of 11 million health workers by 2030, encompassing medical, nursing, and allied health professionals, highlighting a critical challenge for health systems worldwide (World Health Organization 2019). While these shortages are most severe in low‐resource and geographically remote regions (Kharazmi et al. 2024; Naicker et al. 2009; McMahon et al. 2020), countries across all income levels are increasingly struggling to sustain a well‐distributed, adequately resourced, and resilient healthcare workforce (Zhang et al. 2020; Anderson et al. 2021; He et al. 2020). This includes New Zealand (NZ), which, despite being recognised as a high‐performing, publicly funded healthcare system, faces the same mounting workforce pressures (Shuker et al. 2015).

Health workforce challenges are driven by multiple intersecting factors, including an ageing health professional population, urban–rural maldistribution, limited capacity in training and education pipelines, persistent underinvestment in workforce infrastructure, and growing healthcare demands associated with population ageing, multimorbidity, and increasing clinical complexity (Liu et al. 2017; World Health Organization 2016). Of particular concern is the rising rate of health worker attrition, defined broadly as the involuntary or voluntary exit from the workforce (Lopes et al. 2017). Alongside losses from retirement and international migration (Walker and Clendon 2013), a high prevalence of intention‐to‐leave, commonly understood as a worker's self‐reported likelihood or plan to voluntarily exit their current role or profession in the near future, has been identified among practitioners and is established as the most significant predictor of subsequent voluntary turnover (Griffeth et al. 2000). Commonly cited drivers of intention‐to‐leave and subsequent workforce exit include chronic understaffing, excessive workloads, administrative burdens, limited career progression, and insufficient collegial and supervisory support (Roth et al. 2024; Martin et al. 2024). These factors increase the risk of burnout, reduce workforce productivity, compromise care delivery, and lead to adverse patient outcomes, fuelling a cyclical process of workforce instability (Ross 2022).

In NZ, projections from Te Whatu Ora – Health NZ (2023) forecast substantial workforce shortfalls by 2032, including an estimated 14.0% deficit in the medical workforce and 18.0% in the nursing workforce, with additional shortages expected across allied health professions (Te Whatu Ora 2023). NZ's heavy reliance on internationally trained health professionals, which is among the highest in the Organisation for Economic Co‐operation and Development (OECD), renders its health system particularly vulnerable to global competition for skilled labour (OECD 2021). Simultaneously, the emigration of NZ‐trained clinicians, driven by more favourable remuneration, working conditions, and career development opportunities abroad, further challenges domestic retention efforts (Lynch 2008; Yow et al. 2015).

Although the number of national workforce reports, empirical investigations, and professional body commentary on health worker attrition and intention‐to‐leave has increased, these data have not been systematically synthesised. This systematic review and meta‐analysis, therefore, aimed to: (1) estimate the prevalence of attrition and intention‐to‐leave among healthcare workers in NZ; and (2) identify factors associated with these phenomena.

## 2 Methods

### 2.1 Study Design

The development and reporting of this study were guided by the Preferred Reporting Items for Systematic Reviews and Meta‐Analysis (PRISMA) (Liberati et al. 2009) and the Meta‐Analysis of Observational Studies in Epidemiology checklist (Stroup et al. 2000). This review presents the quantitative findings from a systematic review, originally registered as a mixed‐methods review with the International Prospective Register of Systematic Reviews (PROSPERO CRD: 42024590168). Given the volume of studies identified through the search strategy, the quantitative and qualitative findings are reported separately.

### 2.2 Search Strategy

A comprehensive electronic search was conducted in December 2024 and updated in August 2025 across the following databases: CINAHL, Ovid Medline, Cochrane Library (via OVID), PsychINFO, and Scopus. These databases were selected to ensure a broad, systematic coverage of peer‐reviewed literature on workforce issues among registered health professionals in NZ (Lefebvre et al. 2022). All search strategies incorporated controlled vocabulary, as detailed in Table S1.1. To supplement these database results, the first 100 Google Scholar search results were reviewed for inclusion (Table S1.2). A targeted manual search of grey literature was conducted using Google, with a primary focus on workforce reports published by relevant regulatory bodies and professional associations. Sources were evaluated for relevance and included on a case‐by‐case basis. To ensure methodological rigour, the search strategy was developed in consultation with an Auckland University of Technology (AUT) academic librarian. One reviewer (M.C.) conducted the initial search and exported all retrieved studies into Rayyan, an online systematic review application (Johnson and Phillips 2018). After duplicate removal, two reviewers (M.C., B.S.) independently conducted a reliability exercise on a random sample of 20 studies to ensure consistency in article selection based on titles and abstracts. An a priori agreement threshold of 90% was met with 95% agreement between the two reviewers (95% CI: 75.1%−99.9%). All discrepancies were resolved before the two reviewers proceeded to the full screening phase.

### 2.3 Study Eligibility

Studies were eligible for inclusion if they reported original quantitative data, either from standalone quantitative designs or the quantitative components of mixed‐methods studies. To be included, studies had to examine the prevalence of and/or factors associated with attrition or intention‐to‐leave among participants from any health profession regulated under the NZ Health Practitioners Competence Assurance Act (HPCAA) 2003 (Ministry of Health 2024b). These professions include Chinese medicine services, chiropractic, dentistry, dental hygiene, dental technology, dental and oral health therapy, dietetics, medical laboratory science, anaesthetic technology, medical imaging, radiation therapy, medicine, midwifery, nursing, occupational therapy, optometry, optical dispensing, osteopathy, paramedic services, pharmacy, physiotherapy, podiatry, psychology, and psychotherapy. Participants may be currently or previously employed in the public or private sectors across primary, secondary, or tertiary healthcare settings. However, studies focusing solely on nonregistered or unregulated healthcare workers were excluded to maintain focus on HPCAA‐regulated professions, for which workforce standards and data reporting are consistent and comparable across studies. No publication date restrictions were applied to ensure comprehensive coverage of available research within the NZ context, where relevant workforce data remain limited, and to capture historical perspectives on workforce attrition and intention‐to‐leave.

Studies were excluded if they reported aggregated attrition or intention‐to‐leave data for professions not regulated under the HPCAA 2003 or for health professionals practising outside NZ. Additionally, studies that limited recruitment to only participants who had left the profession or had an intention‐to‐leave were excluded. Non‐English language publications, case reports, conference abstracts, and studies lacking original research were also excluded. In cases where multiple grey literature reports by the same author were published across successive years (e.g., annual workforce reports and surveys), only the most recent version was included. Full texts of the studies deemed potentially eligible based on title and abstract screening were independently reviewed by two reviewers (M.C., B.S.) against the predefined eligibility criteria. Any discrepancies were resolved through discussion and consensus.

### 2.4 Quality Assessment

The methodological quality of the included studies was evaluated using the Mixed‐Methods Appraisal Tool (MMAT) (Hong et al. 2018). Although this review reports only quantitative findings, the MMAT was selected for its ability to provide a single, standardised framework for appraising a range of study designs. While grounded in mixed‐methods theory, the MMAT incorporates robust, design‐specific criteria for quantitative studies, including randomised controlled trials, nonrandomised quantitative studies, and quantitative descriptive research (Hong et al. 2018). Its use enables consistent, comparable assessment of methodological quality across all included studies. The MMAT comprises 27 methodological criteria; however, for each study, only the relevant category(s) were assessed. Each criterion was rated on a nominal scale as “yes” (criterion met), “no” (criterion not met), or “unclear” (insufficient information). Prior to independent quality assessment, two reviewers (M.C., B.S.) conducted a reliability exercise on a random sample of five studies (≈15% of the included articles) to ensure consistent application of the assessment criteria. Of the five studies, three were appraised with full agreement, while the remaining two contained minor discrepancies that were discussed and resolved by consensus. This resulted in an observed agreement rate of 60% (95% CI: 14.7%−94.7%). Although no a priori threshold was set, the purpose of this exercise was to ensure both reviewers were aligned with their interpretation of the criteria before proceeding. Following this exercise, both reviewers independently appraised all included studies. Any further discrepancies were resolved through discussion and consensus.

### 2.5 Data Extraction

Data from all included studies were extracted by a single reviewer (M.C.) using a standardised Microsoft Excel spreadsheet. Extracted information comprised study characteristics (including first author surname, publication year, recruitment year, research design, data collection methods, healthcare profession, and measures of attrition and/or intention‐to‐leave), participant characteristics (sample size, healthcare setting, practice experience, gender, age, and geographical location), and study outcomes related to attrition and intention‐to‐leave (prevalence and associated factors).

### 2.6 Meta‐Analyses

#### 2.6.1 Aim 1: Pooled Prevalence of Attrition and Intention‐to‐Leave

Meta‐analyses were conducted to estimate pooled prevalence rates of attrition and intention‐to‐leave. Given substantial variation in workforce dynamics across professions, the primary analyses focused on per‐profession pooled prevalence estimates to provide more meaningful, context‐specific insights. These estimates were derived separately for each profession where sufficient data were available. In addition to per‐profession analyses, an overall pooled prevalence estimate was calculated to provide a broad national‐level indicator of workforce retention trends. However, this cross‐profession estimate should be interpreted with caution, as it reflects an average across studies that are disproportionally weighted towards professions with greater research representation.

Only studies reporting outcomes as raw counts (*n*) or proportions (%) were included, allowing extraction of data for the comparator and outcome groups. Comparator groups were defined as participants who had not left their profession and/or had no intention of doing so, while outcome groups comprised individuals who had either left the profession or indicated an intention‐to‐leave. Studies presenting attrition and/or intention‐to‐leave data solely as means, medians, or ranges were excluded from the meta‐analyses. When outcome data were presented graphically, digital callipers were used to retrieve the relevant values. For studies reporting attrition and/or intention‐to‐leave at multiple yearly intervals, the interval contributing the largest participant sample size was selected for inclusion in the meta‐analyses. If sample sizes were equivalent across yearly intervals, data from the most recent yearly interval were used.

Publication bias was evaluated visually using funnel plots and statistically using Egger's regression test for asymmetry. A statistically significant Egger's test result (*p* < 0.05) was interpreted as potential evidence for publication bias.

#### 2.6.2 Aim 2: Factors Associated with Attrition and Intention‐to‐Leave

To examine factors associated with attrition and intention‐to‐leave, data from all reported yearly intervals were incorporated into meta‐analyses assessing temporal trends. Where the recruitment year was unspecified, the publication year was used as a proxy. To enhance comparability across studies, data reported using different measures, categories, or scales were converted or grouped into standardised formats prior to analysis.

Subgroup analyses were defined a priori based on known factors influencing health workforce attrition and intention‐to‐leave identified in the literature (Roth et al. 2023; Martin et al. 2024; Ross 2022). These included demographic factors (e.g., age, gender, ethnicity, geographic placement), psychological variables (e.g., job satisfaction, burnout), and organisational factors (e.g., workload, healthcare setting, access to support, access to professional development). Additional factors were considered post hoc, where sufficient prevalence data were available across both the comparator and outcome groups. Only factors that reported prevalence data for both comparator and outcome groups were eligible for inclusion in the meta‐analyses. Variables were categorised into subgroups based on consistent thresholds or classifications across studies. For attrition, individuals were categorised into young (ages 20–44 years), middle‐aged (45–55 years), and older (>55 years). For intention‐to‐leave, age was grouped as young (<40 years), middle‐aged (40–54 years), and older (>55 years). Where data permitted, meta‐regressions were conducted to examine differences in attrition and intention‐to‐leave. Subgroup analyses were then conducted to pool estimates within each category and to assess significance between subgroups. Subgroup classifications were applied at the study level, based on aggregate characteristics reported by each study. Mixed‐effects models were applied, which assumed random effects within subgroups and fixed effects between them. A common estimate of between‐study heterogeneity was assumed across all subgroups. Overall heterogeneity was quantified using the I^2^ statistic and reported for all analyses in which values exceeded 0% (Lefebvre et al. 2022). Sensitivity analyses were conducted manually by sequentially excluding individual studies within relevant subgroup categories, including recruitment decade, profession, age group, and healthcare sector, to assess their influence on the pooled prevalence estimates. All meta‐analyses were conducted in R (version 4.5.0) using the *meta* package (version 8.1.0). The metaprop function was used to pool proportions, applying the default arcsine transformation method for variance estimation. Prevelence estimates are presented in the forest plots as pooled proportions, with corresponding percentage values reported in the narrative text to facilitate ease of interpretation.

## 3 Results

### 3.1 Search Results

**FIGURE 1:**
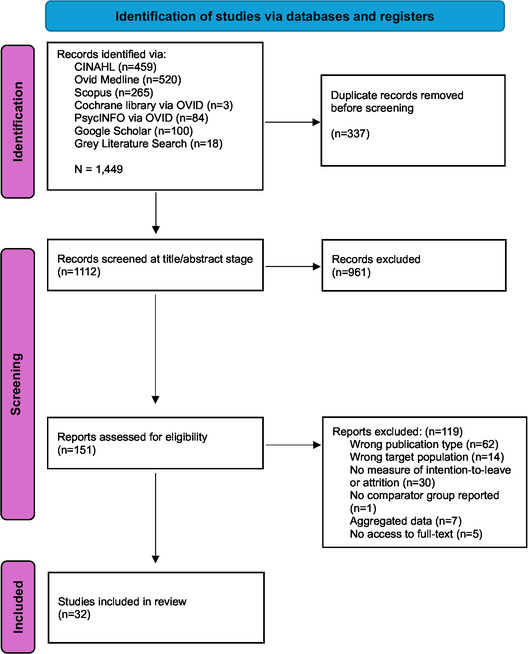
PRISMA flowchart of the systematic review process.

A total of 1,449 studies were identified through database and grey literature searches (Figure 1). After removing duplicates, 1,112 records remained for title and abstract screening. Of these, 151 full‐text articles were assessed for eligibility. Following the full‐text screening, 119 were excluded, with reasons documented in Figure 1. This process resulted in 32 studies retained for inclusion in the final review.

### 3.2 Study and Participant Characteristics

Table 1 summarises the key study and participant characteristics. The majority of studies were published between 2010 and 2019 (*n* = 15), with only one published prior to 2000. Most employed a cross‐sectional design (*n* = 24), while the remainder used retrospective or longitudinal methodologies. Sample sizes varied considerably, ranging from 66 to 19,075 participants. The study populations were predominantly female, with reported mean ages ranging from 25.2 to 56.4 years. Nurses were the most frequently studied professional group (*n* = 15), followed by medical doctors and specialist physicians (*n* = 8) and pharmacists (*n* = 3). Other represented professions included psychologists, physiotherapists, midwives, radiation therapists, occupational therapists, and medical laboratory professionals. Studies were conducted across diverse healthcare settings, encompassing a range of clinical contexts and levels of care. Of the included studies, six reported attrition, 23 investigated intention‐to‐leave, and three explored both outcomes.

**TABLE 1:** Study and participants’ characteristics of included studies (*n* = 32).

					**Prevalence** b		
**1** ^**st**^ **Author, Year**	**Data collection method**	**Profession**	**Sample size** a	**Gender**	**Intention‐to‐leave**	**Attrition**	**Factors associated**
Association of Salaried Medical Specialists (2022)	Online survey	Doctors; Dentists	1,594	F; 44.7% M; 53.8%	18.0% (within 5 years)	—	Dissatisfaction with work arrangement, responsibility, recognition, physical work conditions, work hours
Bradley et al. (2024b) a	Online survey	Pharmacists	416	F; 73.0% M; 24.0%	33.0% (within 5 years)	—	NR
Bradley et al. (2024a) b	Online survey	Pharmacists	345	F; 73.0% M; 24.0%	—	22.0%	Unmanageable stress and burnout
Chambers and Frampton (2022)	Online survey	Psychiatrists	342	F; 45.4% M; 49.7%	23.0% (within 6–12 months)	—	Burnout, stress, poor support, poor job resourcing, covering of caseloads, dissatisfaction
Clendon and Walker (2011)	Online survey	Nurses	674	F; 89.9% M; 10.1%	10.3% (within 12 months)	—	NR
Domett (2024)	Online survey	Doctors	1,300	F; 87.0% M;12.0%	14.0% (within 12 months)	—	Retirement, overseas work
Finlayson et al. (2007)	Mailed questionnaire	Nurses	4,603	F; 93.1%	33.8% (within 12 months)	—	Younger age
Jiang et al. (2023)	Online survey	Nurses	294	F; 89.2%	NR	—	Incivility (coworker, doctor, patient), need for belongingness, competence, and autonomy
Kalliath and Beck (2001)	Questionnaire	Nurses	250	—	NR	—	Emotional exhaustion, depersonalisation, low supervisory support
Lau et al. (2004)	Postal survey	Psychiatrists	159	F; 35.8% M; 64.2%	23.3% (within 10 years)	—	Low pay, poor professional support, low opportunities, stress/burnout, family proximity
Anatomical Pathology Scientific and Technical Workforce Situation Analysis (2014)	Online survey	Medical laboratory professionals	66	F; 73.4%	8.0% (within 5 years)	—	Retirement, career change
Medical Council of New Zealand (2024)	Online survey; Registration data	Doctors	19,075	F; 28.9% M; 51.0%	—	NR	Younger and older age, early career stage, international graduate origin
Moloney, Boxall, et al. (2018) a	Online survey	Nurses	2,876	F; 93.9% M; 6.1%	NR	—	Burnout, high workload, work–life conflict, emotional demands, low engagement, low support, low autonomy
Moloney, Gorman, et al. (2018) b	Online survey	Nurses	2,175	F; 93.9% M; 6.1%	22.6% (unspecified)	—	Low job satisfaction, low career orientation, low work engagement, burnout
Moloney et al. (2024)	Online survey	Nurses	231	F; 98.5% M; 1.5%	NR	—	Low vitality, low organisational support, ineffective leadership
Ng et al. (1991)	Questionnaires	Nurses	1,002	F; 96%	16.2% (within 15 months)	—	Marital status, training, prior jobs
North et al. (2013)	Registration data	Nurses	1,236	F; 94.0% M; 6.0%	—	25.6%	Younger age, lack of postgraduate qualification, practice setting
North et al. (2014)	Registration data	Nurses	12,606	F; 94.0%	—	25.0%	Older age, working part‐time, practice setting
Pick et al. (2008)	Postal survey; Online survey	Ophthalmologists	121	—	23.1% (within 5 years)	—	NR
Price et al. (2016)	Online survey	Nurses	138	F; 96.4% M; 3.6%	37.0% (within 12 months)	7.2%	Attrition: Migration to Australia, lack of postgraduate qualifications, mismatch with preferred working area
Pharmaceutical Society of New Zealand (2024)	Online survey	Pharmacists	431	—	49.2% (within 5 years)	—	Working in community settings
Psychology Workforce Task Group (2017)	Online survey	Psychologists	643	F; 77.0% M; 23.0%	40.0% (within 5 years)	—	Poor pay/conditions, lack of resources, low value, distrust of management, staff conflict, poor communication, no advancement
Ram et al. (2025)	Online survey	Nurses	323	F; 85.0%	15.0% (unspecified)	—	Family/personal reasons, better job opportunities, location preference, career advancement, familiarity with other systems
Ravenswood et al. (2021)	Online survey	Nurses	189	—	33.8% (within 12 months)	—	Stress/burnout, family responsibilities, poor employment conditions
Reid (2019)	Online survey	Physiotherapists	1,207	F; 77.2% M; 22.6%	17.8% (unspecified)	11.3%	Intention to leave: Poor leadership/support, low recognition, lack of role‐clarity, work–life conflict, dissatisfaction, poor communication, poor job‐fit, low workplace commitment, CPD burden, inadequate salary and job prospects. Attrition: predictability and recognition, quality of leadership and recognition, quality of leadership and support, role clarity and recognition, meaning of work and workplace commitment, CPD interference and family plans, CPD interference and ease of completing hours, APC costs and family plans, work prospects and salary adequacy
The Royal New Zealand College of General Practitioners (2023)	Online survey	Doctors	3,281	F; 58.0% M; 42.0%	9.0% (within 5 years)	—	Burnout, younger age, gender, practice ownership, male gender
Shelker et al. (2014)	Registration data	Doctors	3,865	Auckland: M; 46.1% Otago: M; 46.7%	—	25.9%	Entry pathway into medical school
Stokes and Dixon (2018)	Online survey	Occupational therapists	1,059	F; 93.0% M; 7.0%	—	NR	Parental leave, poor working conditions, career change, overseas work
Taylor and Oetzel (2020)	Online survey	Radiation therapists	362	F; 87.0% M; 13.0%	33.0% (unspecified)	—	Workload dissatisfaction, poor professional development, younger age, low pride in role, poor role variety
Wakelin and Skinner (2007)	Phone survey	Midwives	94	NR	54.3% (within 5 years)	25.5%	Intention to leave and attrition: Exhaustion, no personal time, medio‐legal anxiety
Walker et al. (2016)	Online survey	Nurses	562	F; 63.0% M; 27.0%	12.0% (unspecified)	—	NR
Walker (2017)	Online survey	Nurses	628	F; 95.0% M; 5.0%	16.7% (within 5 years)	—	NR

a Sample size of participants in which attrition and/or intention‐to‐leave were assessed.

b For studies examining both outcomes, ITL percentages were calculated based on total sample remaining after attrition; NR = Not reported; Unspecified = no exact timeframe of planned exit reported; CPD = Continued Professional Development; NZ = New Zealand; ASMS = Association of Salaried Medical Specialists; NZWIM = New Zealand Women in Medicine; RNZC*P* = Royal New Zealand College of General Practitioners.

Data collection was predominantly conducted through online surveys. Attrition was assessed either via administrative tracking of professional registration records or through self‐reported survey responses (Table S2)**.** Intention‐to‐leave was primarily measured using Likert‐scale items, binary response questions, and validated multi‐item tools that assessed participants’ likelihood or agreement with leaving their role or profession, often within a specified timeframe (Table S3). As outlined in Table S3, the reported timeframes for the planned exit ranged from 6 months to 10 years, with some studies not specifying a timeframe. However, most studies focused on a 5‐year horizon; this variability was acknowledged as a limitation in interpreting pooled intention‐to‐leave estimates. Where possible, study outcomes were dichotomised and included in the meta‐analysis. The considerable variation in the methods used to capture these outcomes limited comparability and contributed to heterogeneity in the findings.

### 3.3 Quality Assessment

**FIGURE 2:**
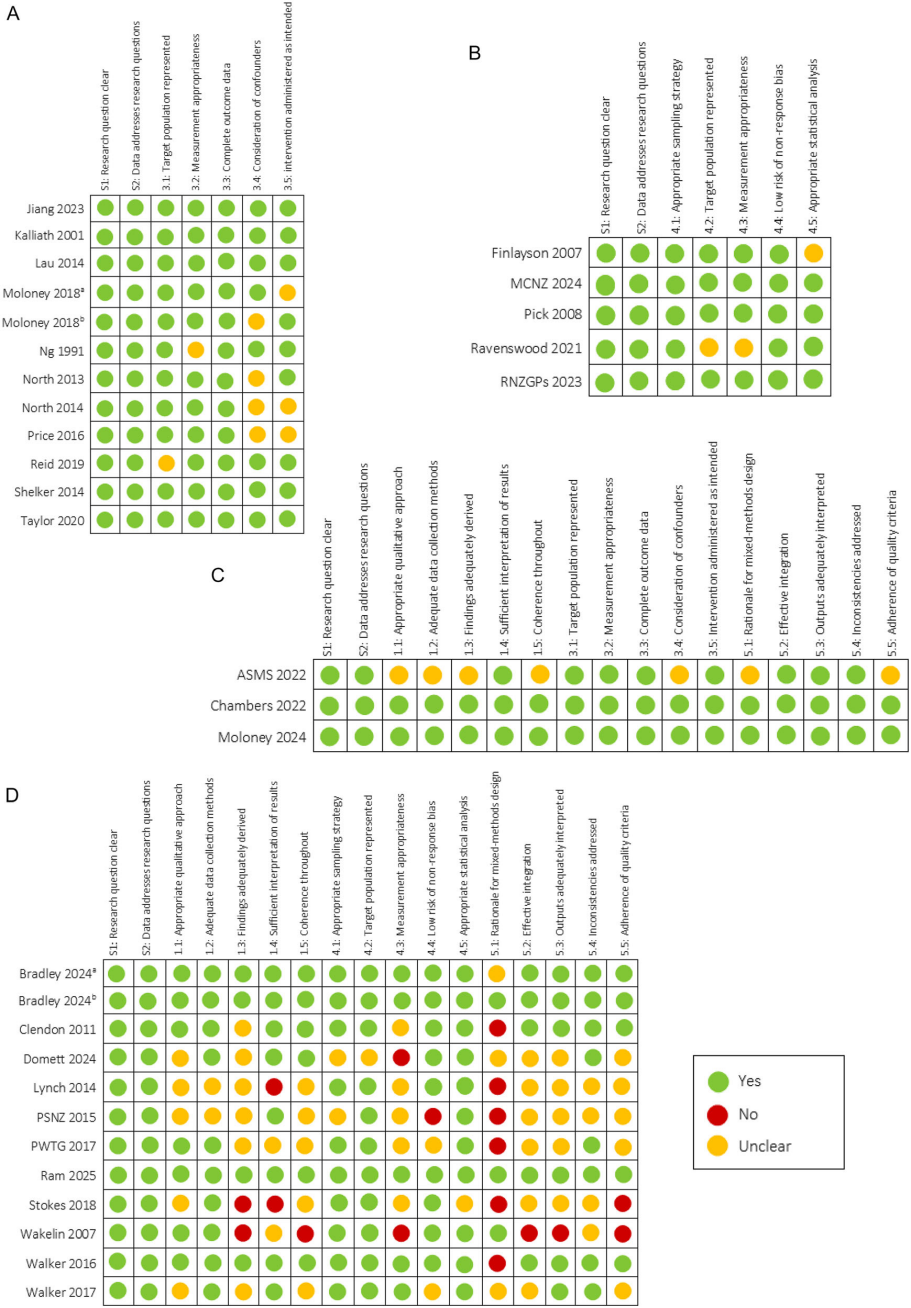
Quality assessment of included studies using the MMAT. (A) Nonrandomised quantitative designs; (B) descriptive quantitative designs; (C) mixed‐methods nonrandomised designs; (D) mixed‐methods descriptive designs.

The quality assessment of the included studies revealed variable methodological rigour across study types (Figure 2). Studies assessed under the quantitative nonrandomised appraisal category are displayed in Figure 2A, and quantitative descriptive studies are presented in Figure 2B. Mixed‐methods (nonrandomised) and mixed‐methods (descriptive) studies are presented in Figure 2C,D, respectively. All included studies clearly articulated their research questions and collected data appropriate for addressing them. Study limitations included occasional unclear reporting around confounder control and measurement fidelity. Mixed‐methods (descriptive) studies showed the greatest variability in methodological quality. Several studies have demonstrated weaknesses in meeting qualitative quality standards, particularly in the rigour of data analysis, clarity of interpretation, and alignment between data and conclusions. Additionally, most did not provide a clear rationale for employing a mixed‐methods approach, and the integration of qualitative and quantitative components was often inadequately explained.

### 3.4 Prevalence of Attrition

#### 3.4.1 Attrition Prevalence by Profession

Attrition prevalence varied across the nine included studies (Table 1). Among nurses, rates ranged from 7.2% (Price et al. 2016), to approximately 25% (North et al. 2013, 2014). Midwives also experienced high attrition, with Wakelin (2007) reporting 25.5% among lead maternity care midwives (Wakelin and Skinner 2007). Comparable levels were observed among doctors, with Shelker et al. (2014) documenting a rate of 25.9% (Shelker et al. 2014). Pharmacists showed moderately lower attrition (22.0%) (Bradley et al. 2024a), while physiotherapists reported the lowest rate of 11.3% (Reid 2019).

#### 3.4.2 Pooled Attrition Prevalence by Profession

**FIGURE 3:**
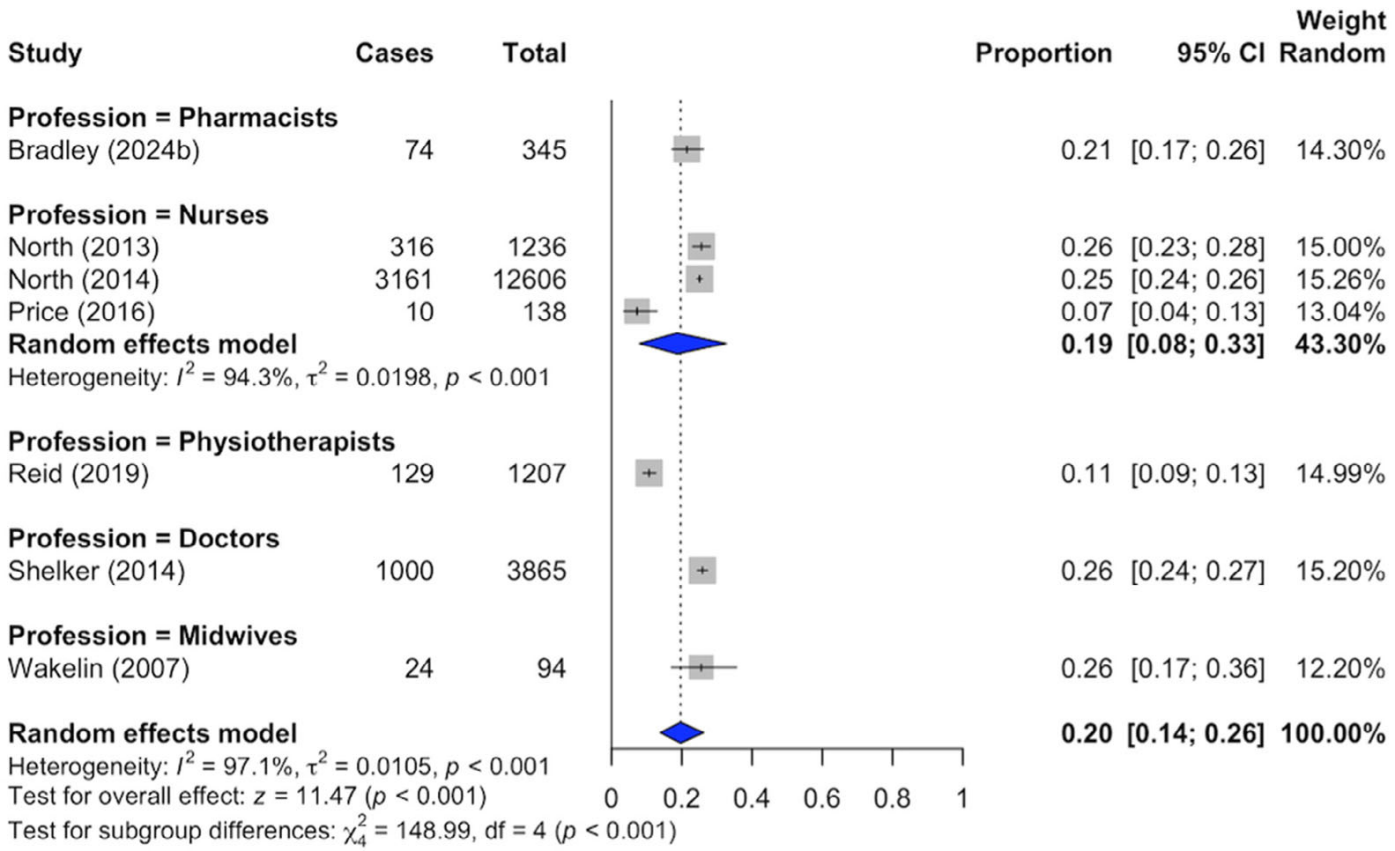
Pooled attrition prevalence estimates by profession. *Note:* Corresponding values are reported as percentages in‐text.

Random‐effects meta‐analyses estimated a pooled attrition prevalence for nurses, the only profession represented by multiple studies (Figure 3). The pooled attrition rate among nurses was 19.0% (95% CI: 8.0%−33.0%).

#### 3.4.3 Cross‐Profession Pooled Attrition Prevalence

To provide a broad national indicator, data from eight studies were combined to calculate a pooled estimate across all professions (Figure S1). Using a random‐effects model, the pooled attrition prevalence was 19.0% (95% CI: 13.0%−25.0%). This estimate should be interpreted cautiously, as it reflects an average across heterogeneous professional groups and serves as an indicative rather than a definitive cross‐profession estimate.

### 3.5 Prevalence of Intention‐to‐Leave

#### 3.5.1 Intention‐to‐Leave Prevalence by Profession

The prevalence of intention‐to‐leave varied considerably across the 26 included studies (Table 1). The lowest rate was reported by Anatomical Pathology Scientific and Technical Workforce Situation Analysis (2014), with 8.0% of medical laboratory professionals indicating an intention‐to‐leave. This was followed by 9.0% among general practitioners (Medical Council of New Zealand 2024), while other physician specialities, such as psychiatrists, reported rates as high as 23.0% (Chambers and Frampton 2022; Lau et al. 2004). Among nurses, estimates ranged widely from 10.3% (Clendon and Walker 2011) to 37.0% (Price et al. 2016). Physiotherapists showed comparatively lower rates (17.8%) (Reid 2019), whereas radiation therapists (33.0%) (Taylor and Oetzel 2020) and psychologists (40.0%) (Psychology Workforce Task Group 2017) demonstrated higher levels. Pharmacists reported 33.0% and 49.2% (Bradley et al. 2024b; Pharmaceutical Society of New Zealand 2024), and midwives exhibited the highest prevalence at 54.3% (Wakelin and Skinner 2007). Notably, the timeframe used to assess intention‐to‐leave varied across studies, ranging from short‐term intentions to longer or unspecified periods. This methodological heterogeneity likely contributed to the wide variation in reported prevalence estimates.

#### 3.5.2 Pooled Intention‐to‐Leave Prevalence by Profession

**FIGURE 4:**
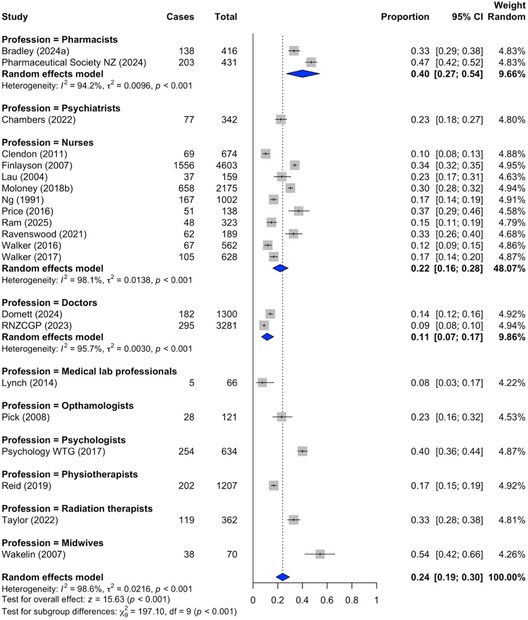
Pooled intention‐to‐leave prevalence estimates by profession. *Note:* Corresponding values are reported as percentages in‐text.

Random‐effects meta‐analyses estimated pooled intention‐to‐leave prevalence by profession (Figure 4). The highest pooled rate was observed among pharmacists (40.0%, 95% CI: 27.0%−54.0%), followed by nurses (22.0%, 95% CI: 16.0%−28.0%). Doctors exhibited the lowest pooled prevalence (11.0%, 95% CI: 7.0%−17.0%).

#### 3.5.3 Cross‐Profession Pooled Intention‐to‐Leave Prevalence

Data from 22 studies were combined using a random‐effects model to estimate the overall pooled prevalence of intention‐to‐leave among healthcare professionals (Figure S2). The pooled prevalence was 24.0% (95% CI: 19.0%−29.0%). As with attrition, this estimate should be interpreted as a general indicator given the heterogeneity and uneven representation across professional groups.

### 3.6 Factors Associated with Attrition

Factors associated with attrition spanned individual, professional, and organisational domains (Tables 1 and S2). Common individual‐level factors included age and career stage, with both younger and older cohorts variably represented (North et al. 2013, 2014; Medical Council of New Zealand 2024), as well as personal circumstances such as limited time for self‐care and family responsibilities (Reid 2019; Stokes and Dixon 2018). Exhaustion and burnout were key drivers of attrition among early‐career pharmacists (Bradley et al. 2024a) and lead maternity care midwives (Wakelin and Skinner 2007). Professional factors included the absence of a postgraduate qualification (Price et al. 2016; North et al. 2013), insufficient peer or supervisory support, and a perceived lack of recognition (Reid 2019). Organisational contributors encompassed workplace setting, conditions, and hours (Price et al. 2016; North et al. 2014; Stokes and Dixon 2018; North et al. 2012). Two studies identified international migration as a primary driver of attrition (Price et al. 2016; Stokes and Dixon 2018).

#### 3.6.1 Subgroup Analyses of Attrition

Subgroup analysis demonstrated variability in attrition prevalence across demographic, educational, and temporal factors (Table 2). Attrition differed significantly by profession (*p* < 0.001), with midwives and doctors exhibiting the highest rates and physiotherapists the lowest. Individuals without a postgraduate qualification had significantly higher attrition rates than those with postgraduate qualifications (*p* < 0.001). Although not statistically significant, attrition appeared more prevalent among younger workers (*p* = 0.380). Studies conducted in the 2000s reported higher attrition rates than those in the 2010s (*p* = 0.51), and rates were slightly higher among females (*p* = 0.940). Due to the limited number of studies in several subgroups, meta‐regression was not feasible.

**TABLE 2:** Subgroup analyses of attrition among NZ healthcare workers.

Variable	Characteristic	No. studies	Total sample	** Prevalence (95% CI)** **a**	**I** ^**2**^	*P* for heterogeneity	*P* value between group differences
Recruitment	2020s	1	345	0.21 (0.17 to 0.26)	—	—	0.51
Decade	2010s	5	18,052	0.18 (0.11 to 0.27)	98.0%	<0.001	
	2000s	1	94	0.26 (0.17 to 0.36)	—	—	
Profession	Pharmacists	1	345	0.21 (0.17 to 0.26)	—	—	<0.001
	Nurses	3	13,980	0.19 (0.08 to 0.33)	94.3%	<0.001	
	Doctors	1	3865	0.26 (0.24 to 0.27)	—	—	
	Physiotherapists	1	1207	0.11 (0.09 to 0.13)	—	—	
	Midwives	1	94	0.26 (0.17 to 0.36)	—	—	
Age	Younger	2	1092	0.20 (0.04 to 0.44)	98.7%	<0.001	0.380
	Middle	2	6165	0.10 (0.00 to 0.35)	99.6%	<0.001	
	Older	1	7020	0.07 (0.07 to 0.08)	—	—	
Gender	Female	2	2015	0.19 (0.08 to 0.32)	97.9%	<0.001	0.940
	Male	2	324	0.17 (0.02 to 0.45)	95.5%	<0.001	
Postgraduate	Yes	2	295	0.09 (0.06 to 0.13)	0%	0.62	<0.001
Engagement	No	1	1004	0.29 (0.26 to 0.32)	—	—	

a Values presented as proportions (0–1). Corresponding values are reported as percentages in‐text.

#### 3.6.2 Factors Associated with Intention‐to‐Leave

Factors influencing intention‐to‐leave encompassed individual, occupational, and organisational domains (Table 1 and Table S3). Individual factors included age (Finlayson et al. 2007; The Royal New Zealand College of General Practitioners 2023; Taylor and Oetzel 2020) and personal or family circumstances (Wakelin and Skinner 2007; Lau et al. 2004; Ram et al. 2025; Ravenswood et al. 2021). Burnout, occupational stress, and dissatisfaction were consistently identified as key predictors (Reid 2019; Chambers and Frampton 2022; Lau et al 2004; The Royal New Zealand College of General Practitioners 2023; Taylor and Oetzel 2020; Ravenswood et al. 2021; Moloney, Gorman, et al. 2018; Moloney, Boxall, et al. 2018; Association of Salaried Medical Specialists 2022).

Organisational and professional contributors included the lack of recognition, increased responsibility (Reid 2019; Ravenswood et al. 2021; Association of Salaried Medical Specialists 2022), and inadequate support or leadership (Reid 2019; Chambers and Frampton 2022; Lau et al. 2004; Moloney, Boxall, et al. 2018; Kalliath and Beck 2001; Moloney et al. 2024). Other factors included adverse working conditions, high workload, limited advancement opportunities, and practice setting (Price et al. 2016; North et al. 2013; North et al. 2014; Chambers and Frampton 2022; Lau et al. 2004; Psychology Workforce Task Group 2017; Moloney, Gorman, et al. 2018; Moloney et al. 2024). Three studies also linked migration intentions to intention‐to‐leave (Price et al. 2016; Stokes and Dixon 2018; Domett 2024).

#### 3.6.3 Moderator and Subgroup Analyses of Intention‐to‐Leave

Subgroup analyses examined variation in intention‐to‐leave prevalence across demographic, temporal, and healthcare‐sector factors (Table 3). Profession‐specific prevalences differed significantly (*p* < 0.001), with midwives exhibiting the highest prevalence and medical laboratory professionals the lowest. Studies from the 2000s reported significantly higher intention‐to‐leave prevalence than those from the 2010s (*p* = 0.030). Although not statistically significant, younger workers reported a higher prevalence, while middle‐aged workers reported the lowest (*p* = 0.850). No significant differences were found by gender (*p* = 0.940). Practitioners in nonpatient‐facing roles reported a higher prevalence than those in hospital or secondary care settings, though this difference was not statistically significant (*p* = 0.580). Meta‐regression analyses across age, gender, and profession subgroups identified no statistically significant moderator effects.

**TABLE 3:** Subgroup analyses of intention‐to‐leave among NZ healthcare workers.

Variable	Characteristic	No. studies	Total sample	** Prevalence (95% CI)** **a**	**I** ^**2**^	*P* for heterogeneity	*P* value between group differences
Recruitment	2020s	9	8238	0.24 (0.16 to 0.32)	98.5%	<0.001	0.030
Decade	2010s	10	6850	0.19 (0.12 to 0.26)	97.9%	<0.001	
	2000s	4	4953	0.33 (0.20 to 0.47)	89.0%	<0.001	
	1990s	1	1002	0.17 (0.14 to 0.19)	—	—	
Profession	Pharmacists	2	847	0.40 (0.27 to 0.54)	94.2%	<0.001	<0.001
	Psychiatrists	1	368	0.21 (0.17 to 0.25)	—	—	
	Nurses	10	10 453	0.24 (0.18 to 0.32)	98.1%	<0.001	
	Doctors	2	4581	0.11 (0.07 to 0.17)	95.7%	<0.001	
	Medical Laboratory Professionals	1	66	0.08 (0.03 to 0.17)	—	—	
	Ophthalmologists	1	121	0.23 (0.16 to 0.32)	—	—	
	Psychologists	2	1793	0.24 (0.03 to 0.57)	99.5%	<0.001	
	Physiotherapists	1	1207	0.17 (0.15 to 0.19)	—	—	
	Radiation therapists	1	362	0.33 (0.28 to 0.38)	—	—	
	Midwives	1	70	0.54 (0.42 to 0.66)	—	—	
Age	Younger	4	2491	0.21 (0.04 to 0.46)	99.4%	<0.001	0.850
	Middle	3	5536	0.14 (0.04 to 0.28)	99.4%	<0.001	
	Older	2	2017	0.17 (0.00 to 0.56)	99.7%	<0.001	
Gender	Female	4	5505	0.18 (0.09 to 0.28)	99.2%	<0.001	0.940
	Male	4	2605	0.18 (0.12 to 0.26)	92.3%	<0.001	
Healthcare sector	Primary/community	3	982	0.28 (0.09 to 0.52)	98.5%	<0.001	0.580
	Hospital/secondary	2	719	0.25 (0.04 to 0.56)	94.3%	<0.001	
	Nonpatient facing	1	30	0.40 (0.23 to 0.59)	—	—	

a Values presented as proportions (0–1). Corresponding values are reported as percentages in‐text.

### 3.7 Diagnostic Results

**FIGURE 5:**
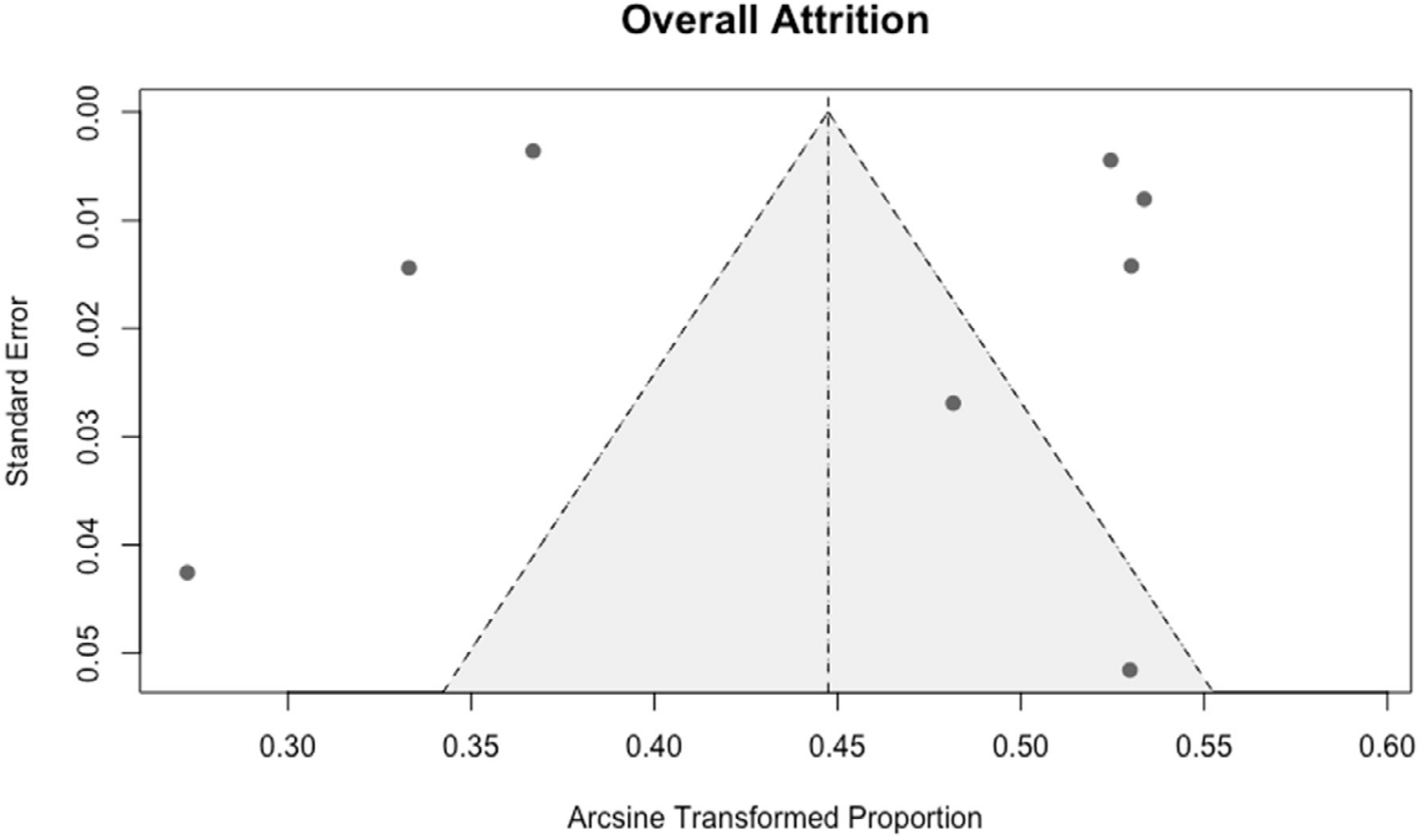
Funnel plot assessing publication bias for attrition prevalence estimates.

**FIGURE 6:**
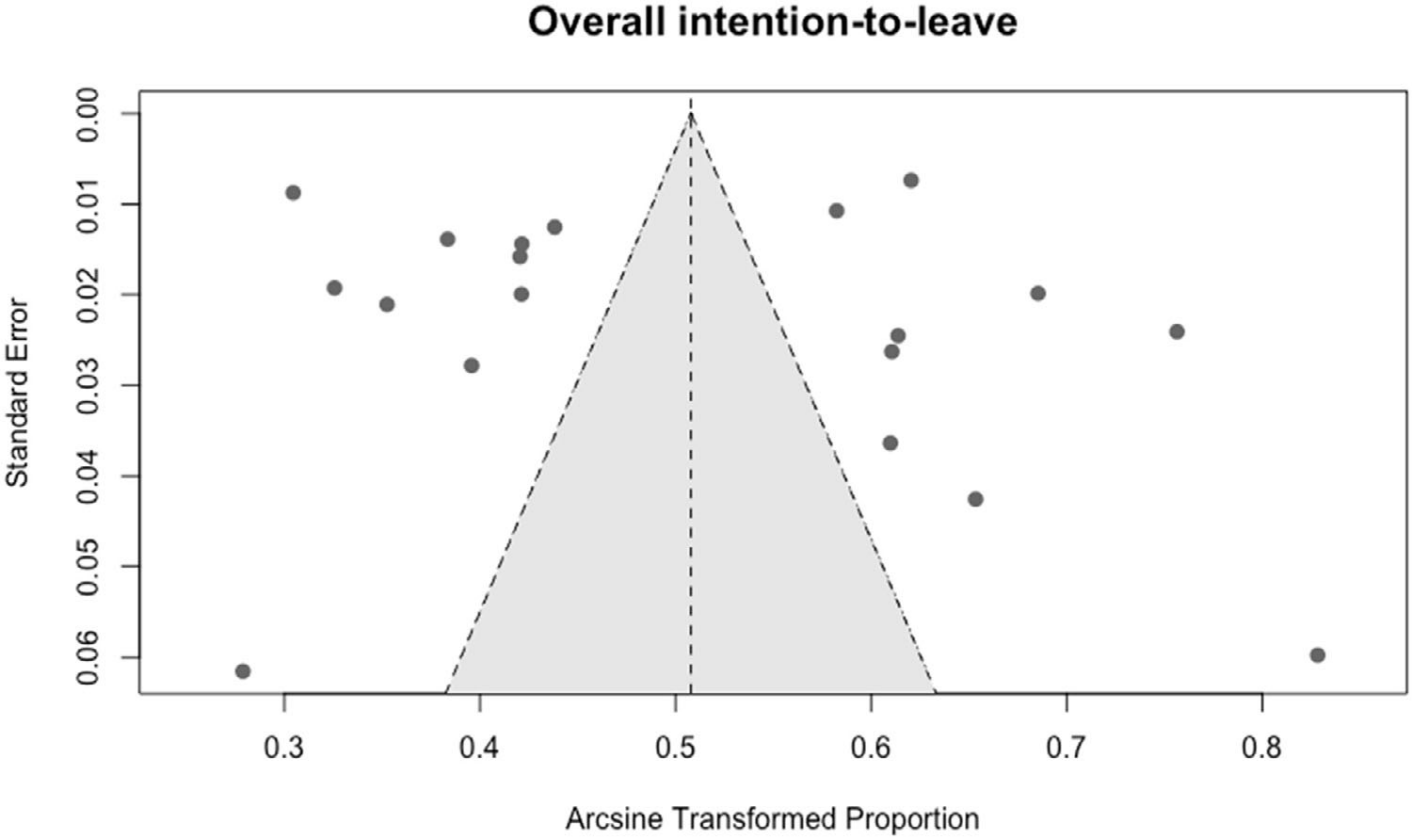
Funnel plot assessing publication bias for intention‐to‐leave prevalence estimates.

Sensitivity analysis, based on the sequential exclusion of individual studies within subgroups, indicated that removing the 1990s study on intention‐to‐leave resulted in a significant between‐group difference (Table 3; *p* = 0.16). All other exclusions did not affect the statistical significance of between‐group differences. Funnel plots were examined to assess potential publication bias (Figures 5 and 6), and supplementary arcsine‐transformed funnel plots against sample size were also generated (Figures S3 and S4). Both sets of plots showed no strong evidence of publication bias, and Egger's regression test for intention‐to‐leave was nonsignificant (*p* = 0.78). Egger's test was not conducted for the pooled attrition estimate due to the limited number of contributing studies (*n* = 8) and reduced statistical power. However, interpretation of these diagnostics should be cautious. Funnel plots and Egger's tests have limited diagnostic accuracy in proportion‐based meta‐analyses; therefore, the absence of visual or statistical asymmetry should not be taken as definitive evidence that publication bias is absent (Hunter et al. 2014).

## 4 Discussion

This review represents the first systematic synthesis of attrition and intention‐to‐leave prevalence, and associated factors, among healthcare professionals in NZ.

### 4.1 Prevalence of Attrition and Intention‐to‐Leave

The professional group emerged as a significant moderator of both attrition and intention‐to‐leave. Midwives consistently demonstrated the highest rates, likely reflecting persistent challenges such as professional isolation, limited organisational support, and under‐resourcing, particularly in rural regions of NZ (Clemons et al. 2020; Daellenbach et al. 2020). The emotionally demanding nature of maternity care, combined with heavy workloads and on‐call duties, may further exacerbate occupational stress and turnover intentions within this group.

Pharmacists also exhibited elevated rates, which may be attributable to role constraints that emphasise dispensing and retail functions over clinical practice, alongside suboptimal working conditions, job dissatisfaction, and psychological stressors, issues well documented in the NZ pharmacy literature (Lam et al. 2023, Health New Zealand Te Whatu Ora 2024). The high turnover intention reported among radiation therapists has concerning implications for cancer care delivery, though this finding is based on a single study and should be interpreted cautiously.

Interestingly, doctors exhibited a paradoxical pattern: high attrition but with low intention‐to‐leave prevalence. The single attrition study in this group examined graduate medical students, and the elevated attrition rate likely reflects overseas migration rather than permanent workforce exit (Shelker et al. 2014). Conversely, the lower intention‐to‐leave prevalence among practising doctors may reflect the substantial personal and financial investment required to enter and remain in medicine, discouraging full withdrawal and instead prompting adjustments such as reduced hours or shifts in clinical settings.

Physiotherapists and medical laboratory professionals reported the lowest attrition and intention‐to‐leave rates, respectively. The predominance of physiotherapists in NZ's private sector may contribute to lower attrition due to greater professional autonomy, control of work–life balance, or higher earning potential (Rehabilitation Therapy ‐ Health New Zealand Te Whatu Ora. n.d.). However, private practice may also introduce distinct stressors, including professional isolation, administrative demands, and market pressures. Similarly, while Te Whatu Ora (Health NZ) currently considers pathology and laboratory services to be relatively well‐resourced, workforce projections suggest emerging capacity challenges (Pathology ‐ Health New Zealand Te Whatu Ora. n.d.). These nuanced interpretations highlight the importance of contextualising findings, given the limited representation of these professions and the influence of study‐specific characteristics, such as urban participant concentration, overrepresentation of experienced practitioners, and limited demographic diversity, on observed outcomes.

Across 32 studies published between 1991 and 2025, pooled prevalence estimates were 19.0% for attrition and 24.0% for intention‐to‐leave. These findings are broadly consistent with global international estimates of healthcare workforce turnover, which range from 18.0% to over 50.0% depending on profession and context (Wu et al. 2024; Shen et al. 2020). In contrast, turnover in other major NZ sectors is notably lower, averaging around 13% in construction (Workforce Dynamics and Demographics | Ministry of Business, Innovation and Employment 2016), 18% in hospitality (Williamson and Harris 2024), and 10% among teachers (Te Whatu Ora 2023). This disparity highlights the comparatively high volatility of the NZ healthcare workforce and underscores the persistent retention challenges facing all health professions.

### 4.2 Factors Associated with Attrition and Intention‐to‐Leave

Temporal trends indicated elevated intention‐to‐leave rates during the 2000s, coinciding with significant health system reforms in NZ, including the establishment of the District Health Boards (DHBs), Primary Health Organisations (PHOs), and the HPCAA 2003 regulatory framework (New Zealand Parliamentary Library 2009). Although these reforms aimed to enhance governance and service integration, workforce planning remained fragmented and misaligned with broader system objectives (Rees 2019). Continued reliance on internationally trained professionals, limited investment in workforce development, and clinician‐management tensions may have exacerbated workforce dissatisfaction during this period (Rees 2019). The Global Financial Crisis (2008–2009) further constrained resources and intensified public sector pressures, contributing to instability (Mills 2010).

Engagement in postgraduate studies was associated with lower attrition rates. While the underlying mechanisms remain unclear, postgraduate study may function as a protective factor by reinforcing professional identity, enhancing clinical capability, and promoting career progression, thereby strengthening organisational commitment and retention (Mohamed et al. 2024). In contrast, some physiotherapists perceived continued professional development (CPD) as contributing to attrition and intention‐to‐leave (Reid 2019). This may reflect perceptions of CPD requirements tied to registration and annual practising certificates as regulatory and administrative burdens rather than meaningful opportunities for professional growth (Hijazen et al. 2023).

While age groups were not statistically significant, younger and early‐career professionals frequently demonstrated higher attrition and intention‐to‐leave, aligning with career development frameworks that identify early stages as vulnerable to unmet expectations and workplace misalignment (Juntunen et al. 2019). In NZ, inadequate supervision, limited advancement opportunities, and professional under‐recognition are common drivers of early‐career attrition (Walker and Clendon 2017; Satchell and Jacobs 2024), further compounded by Trans‐Tasman migration, where Australian opportunities offer high pay and recognition of NZ qualifications (Trans‐Tasman Mutual Recognition Act 1997).

Neither gender nor the healthcare sector was significantly associated with attrition or intention‐to‐leave. While international literature has identified gender‐based differences (Apple et al. 2023; Li et al. 2024), such effects may attenuate when controlling for other determinants. Similarly, the absence of sector‐specific variation may reflect definitional inconsistency or suggest that individual and organisational factors exert greater influence (Heponiemi et al. 2012).

Although data inconsistencies limited meta‐analyses, studies consistently identified personal, professional, and organisational factors contributing to attrition and intention‐to‐leave. Personal demands, such as family responsibilities, were common and often intensified by unpredictable schedules, difficulty balancing roles, and emotional strain (Alzoubi et al. 2024). These pressures may be especially salient to Māori and Pacific health professionals, who frequently carry additional whānau (family) obligations.

Professionally, burnout stems from unmet expectations, insufficient support, and unsustainable working conditions, issues repeatedly voiced in NZ's national media, workforce reports, and professional commentary. The recurrent emphasis of psychological strain underscores its role as a key determinant of attrition, particularly given NZ's ongoing health system pressures (Minister of Health 2024a). Organisational drivers, including inadequate remuneration relative to rising national living costs, limited advancement opportunities, and excessive workloads, highlight systemic issues that undermine workforce morale and stability (Minister of Health 2024a). Variations across healthcare settings may reflect disparities in access to leadership, resources, and peer support, challenges that are especially pronounced in rural and Māori health services (Oetzel et al. 2024; Rural Health Strategy 2023]. In these contexts, culturally safe practice is underpinned by strong leadership and collegial support, both of which are essential for workforce sustainability (Panesar et al. 2021).

### 4.3 Strengths and Limitations

This review provides a comprehensive synthesis of attrition and intention‐to‐leave among healthcare professionals in NZ, spanning more than three decades of published and grey literature. Key strengths include the inclusion of diverse professional groups, integration of grey literature, and the use of meta‐analytic techniques to estimate pooled prevalences and examine subgroup variation. A robust, multidatabase search strategy, combined with independent dual screening, strengthened methodological rigour and reduced the risk of selection bias.

A notable limitation was the lack of differentiation between voluntary and involuntary turnover, which constrained insight into whether workforce exits were primarily driven by individual choice or structural factors. Substantial variation in definitions, measurement instruments, and recruitment periods limited cross‐study comparability and contributed to elevated statistical heterogeneity. Although the inclusion of grey literature broadened the evidence base and enhanced contextual relevance, it also introduced variability in methodological quality and reporting standards. Despite thorough manual screening of grey literature, some relevant sources may have been inadvertently missed, introducing a small risk of selection bias.

Subgroup analyses were further constrained by inconsistent or insufficient reporting, particularly for key variables such as burnout and job satisfaction across distinct healthcare settings. Many HPCAA‐regulated professions were underrepresented in the available literature, limiting the generalisability of the findings across the broader health workforce and highlighting potential vulnerabilities among smaller or less‐studied professions. Although including studies spanning several decades provided a longitudinal perspective on workforce trends, the findings may not fully reflect current practice environments. This was considered when interpreting pooled prevalence estimates, which represent broad trends rather than time‐specific measures.

Importantly, none of the included studies reported outcomes disaggregated by ethnicity, precluding analysis of potential disparities in attrition and/or intention‐to‐leave among indigenous Māori health workers. While Māori practitioners may face unique structural or cultural challenges, strengths such as engagement with Kaupapa Māori health models and culturally grounded leadership may serve as protective factors against workforce loss (Muriwai et al. 2016).

### 4.4 Implications for Policy and Practice

The elevated prevalence of attrition and intention‐to‐leave highlights the need for targeted, profession‐specific retention strategies. Particular attention should be directed toward early‐career professionals and those in high‐stress roles, where vulnerabilities are most pronounced.

There is also an imperative to conduct further research to understand the contributing factors. For example, the sole study examining radiation therapists in NZ reported that exactly one‐third intended to leave the profession. Among midwives, the proportion was considerably higher at 54%, while pharmacists reported a similar concerning figure of 40%. These findings expose structural vulnerabilities within the NZ health workforce, with important implications for workforce sustainability and service delivery.

Strengthening professional recognition and expanding career development opportunities through structured postgraduate pathways, formal mentorship, and transparent advancement frameworks may mitigate and enhance retention. The development of a standardised, psychometrically validated tool tailored to the NZ healthcare context would enable more accurate and consistent measurement of intention‐to‐leave and its determinants. Such a tool would support longitudinal workforce monitoring and support timely, evidence‐informed, and equity‐oriented policy responses. Future research should also prioritise underrepresented professional groups and populations, particularly Māori health professionals, to ensure that workforce strategies are inclusive, culturally responsive, and contextually appropriate across the health sector.

## 5 Conclusion

This review highlights substantial levels of attrition and intention‐to‐leave among NZ healthcare professionals, varying by profession, time period, and engagement in postgraduate education. Burnout, career stage, and organisational stressors emerged as key contributors. Targeted, profession‐specific retention strategies, along with the development of a standardised national tool to measure intention‐to‐leave are essential to enhance workforce stability, inform policy, and support equitable and sustainable healthcare delivery.

## Supporting Information

Additional supporting information can be found online in the Supporting Information section. **Supporting Fig. S1:** Pooled prevalence estimates of overall attrition. **Supporting Fig. S2:** Pooled prevalence estimates of overall intention‐to‐leave (displayed as proportions). Note: Corresponding values are reported as percentages in‐text. **Supporting Fig. S3** Funnel plot of attrition prevalence estimates by transformed proportion and sample size. **Supporting Fig. S4:** Funnel plot of intention‐to‐leave prevalence estimates by transformed proportion and sample size. **Supporting** **Table S1.1:** Search terms used in CINAHL, Ovid Medline, Cochrane library (via OVID), Scopus, and PsycINFO, adapted for each database. **Supporting Table S1.2:** Search strategy used in Google Scholar. **Supporting Table S** **2:** Summary of studies examining attrition (n = 9). **Supporting Table S** **3:** Summary of studies examining intention‐to‐leave (n = 26).

## Funding

This study was supported by Auckland University of Technology, New Zealand (Faculty of Health and Environmental Sciences Summer Studentship Research Award 2024/2025; Vice Chancellor's Doctoral Scholarship).

## Conflicts of Interest

The authors declare no conflicts of interest.

## Supporting information

Supplementary Material
