# Supplementary material for: The Prevalence and Factors Associated with Workforce Attrition and Intention‐to‐Leave Among Healthcare Workers in New Zealand: A Systematic Literature Review and Meta‐Analysis
Source: J R Soc N Z. 2026 Feb 18;56(1):e70025. doi: 10.1002/snz2.70025 (PMC12964993; doi:10.1002/snz2.70025)
Supplement: Supplementary file 1 — Supplementary Material [file SNZ2-56-e70025-s001.pdf]

Supplementary Figure 1: Pooled prevalence estimates of overall attrition

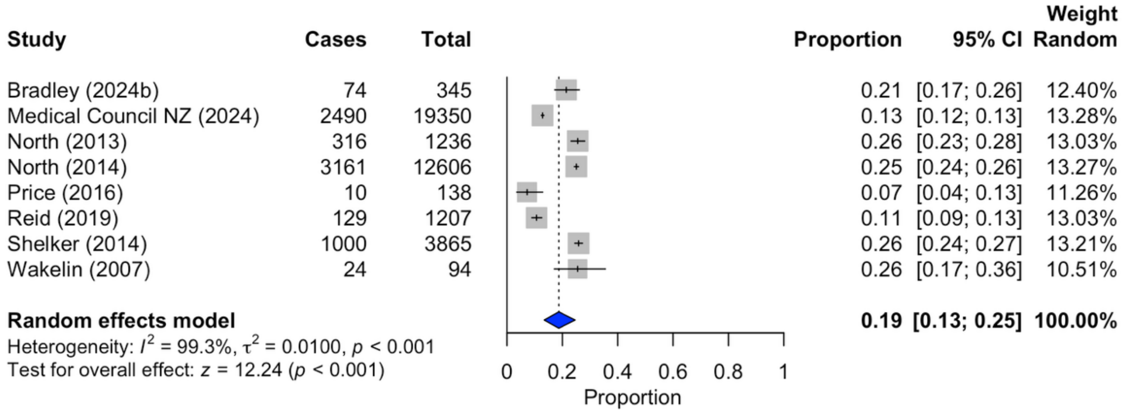

Supplementary Figure 2: Pooled prevalence estimates of overall intention-to-leave (displayed as proportions)

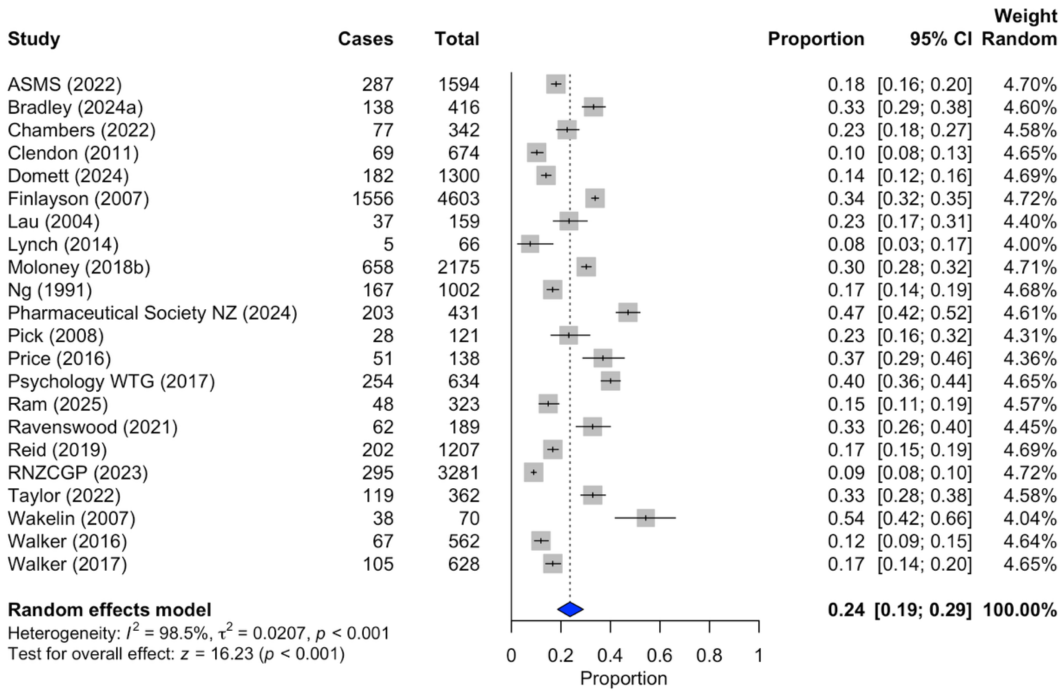

Note: Corresponding values are reported as percentages in-text.

Supplementary Figure 3 Funnel plot of attrition prevalence estimates by transformed proportion and sample size

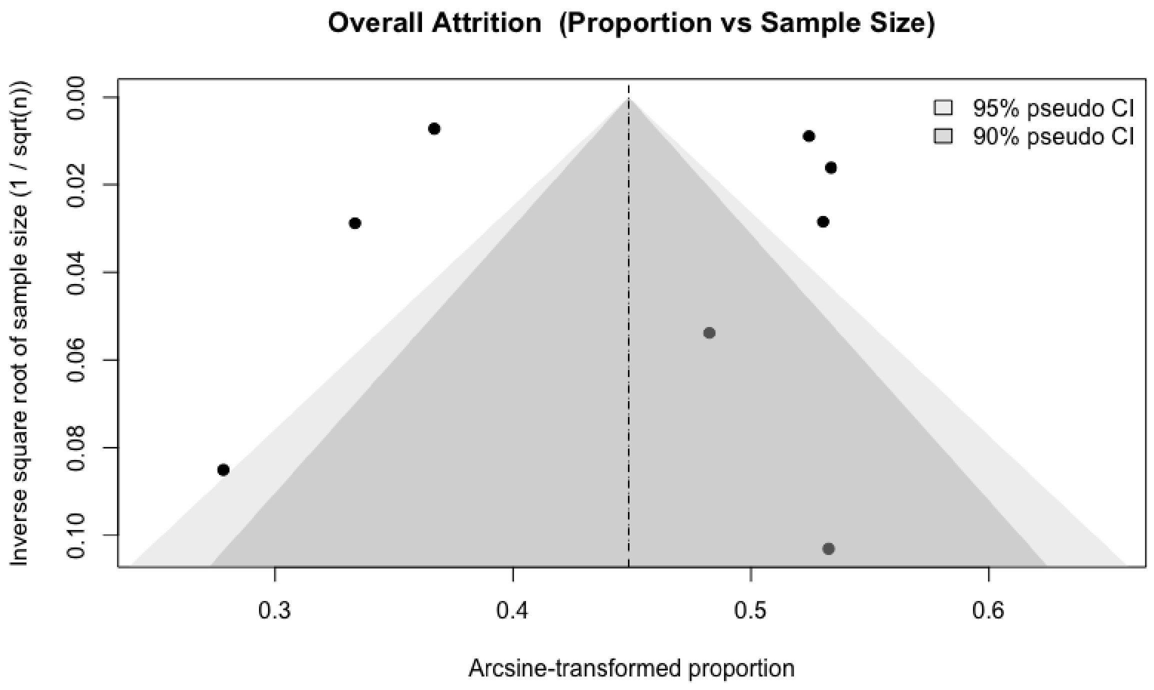

Supplementary Figure 4: Funnel plot of intention-to-leave prevalence estimates by transformed proportion and sample size

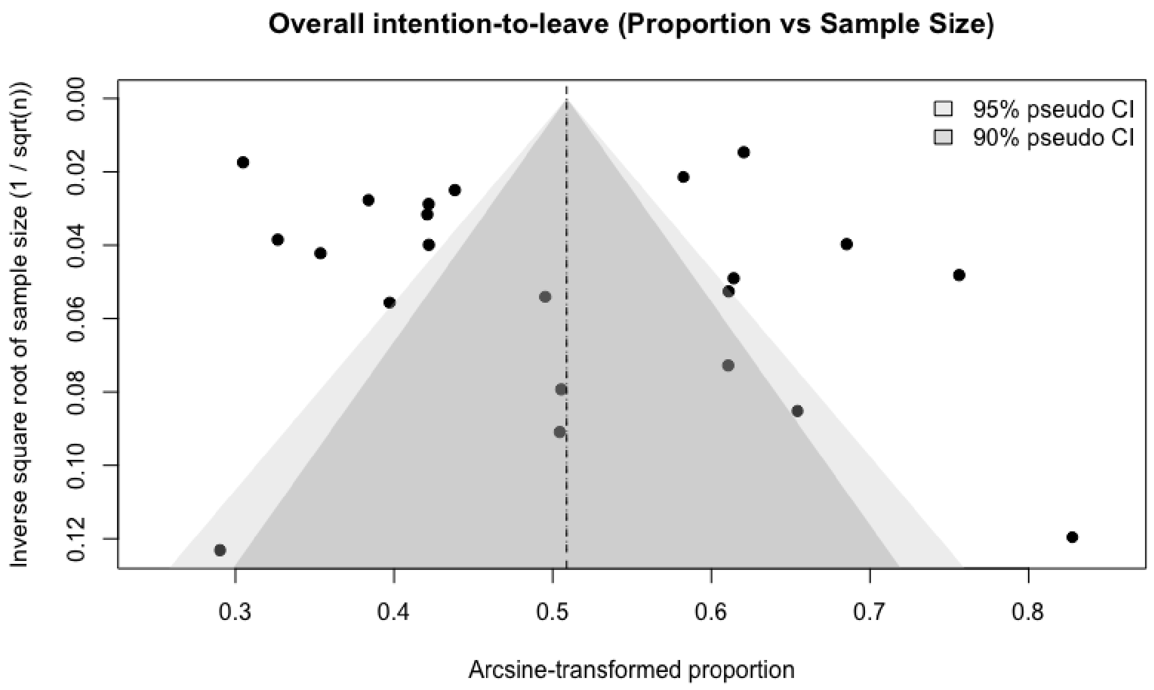

Supplementary Table 1.1: Search terms used in CINAHL, Ovid Medline, Cochrane library (via OVID), Scopus, and PsycINFO, adapted for each database

1	(Attrition OR shortage OR retention OR turnover OR exit OR leav* OR migration OR retain* OR recruitment OR quit*) 6[1] (Staff* OR worker* OR employ* OR clinician OR practitioner* OR professional* OR personnel)
2	"allied health*" OR ambulance OR anaesthes* OR anesthes* OR cardi* OR "chinese medicine" OR chiropract* OR clinic* OR dental OR dentist* OR derm* OR dietetic* OR dietitian OR "dispensing optician" OR doctor* OR "emergency care" OR "general practi*" OR gynae* OR gyne* OR health* OR hospi* OR hospital OR "intensive care" OR "magnetic resonance imaging technolog*" OR maxillofacial OR medic* OR "medical spec*"OR "mental health" OR midwi* OR musculoskeletal OR neuro* OR "nuclear medicine technolog*" OR nurs*OR obstet* OR "occupational therap*" OR oncolo* OR ophthal* OR optometr* OR oral OR ortho* OR orthodontic OR osteopath* OR otolary* OR paediat* OR pain OR palliative OR paramedic* OR path* OR pediat* OR pharmac* OR "physical therap*" OR physician* OR physio* OR podiatr* OR practitioner OR prehospital OR "primary care" OR psych* OR psycholog* OR psychotherap* OR "radiation therap*" OR radio* OR rehab* OR "rural health" OR "secondary care" OR sonographer OR surg* OR "tertiary care" OR "urgent care"
3	"New Zealand*" OR NZ OR Aotearoa OR Maori OR Māori OR Australasia OR Oceania
4	1 AND 2 AND 3

[1]Proximity searching command

Supplementary Table 1.2: Search strategy used in Google Scholar

(Attrition OR shortage OR retention OR turnover OR exit) AND (health OR clinician OR practitioner) AND ("New Zealand" OR NZ OR Aotearoa OR Maori OR Māori OR Australasia OR Oceania)

Supplementary Table 2: Summary of studies examining attrition (n=9)

1st Author, Year	Profession and setting	Sample size[a]	Measurement instrument/methods	Significance of the factors related to attrition	Percentage of reported attrition	Inclusion in meta-analysis
Bradley, 2024[b] [32]	Early-career pharmacists from public and private sectors; 2019	345	Multi-choice options on support accessed during times of unmanageable stress and burnout, including the option 'I left my job'.	Attrition was exclusively related to unmanageable stress/burnout	22.0%	Y
Medical Council NZ, 2024 [35]	Doctors; 2023/2024	19,075	Registration and workforce survey data.	Doctors under 30 and those over 65 years and are more likely to leave compared to those aged between 36 and 55. Doctors are more likely to leave in their first 10 years of practice. Among	NR	N[b]

				international medical graduates, those trained in Africa, the Middle East, and Asia have a higher retention rate than those trained in the Americas, the UK, and Oceania. Among all international medical graduates, ≈40.0% leave after one year, and ≈60.0% after 2 years compared to NZ trained graduates with ≈1.0% and ≈5.0% attrition, respectively.		
North, 2013 [28]	Newly registered NZ graduate nurses; 2005/2010	1,236	Tracking of nursing registration through the Nursing Council of NZ's registration data set from 2005 to 2010. 'Separations' refers to instances where newly registered nurses at baseline were no longer practicing in the NZ nursing workforce during the five years following initial registration.	Nurses aged 20-24 had the highest separation rates (31.5%), and was significantly lower among nurses in their 30s ($p=0.007$). Nurses who gained a postgraduate tertiary qualification within 5 years had significantly lower separation rates (10.0%) compared to those who did not (29.0%)($p<0.001$). Attrition was lowest in mental health (40.0%) and highest in continuing care (90.0%)($p<0.001$). Non-significant factors included ethnicity, gender, urban versus rural practice, and work hours.	25.6%	Y
North, 2014 [29]	Nurses aged ≥50 years; 2006/2011	12,606	Tracking of nursing registration through the Nursing Council of NZ's registration data set from 2006	Permanent separation rates rose sharply from age 64. Nurses employed outside	25.0%	Y

			to 2011. The term ‘permanent separations’ refers to those who left the workforce and did not return by the end of the five year study period.	the DHBs (29.0%) had significantly higher separation rates than those employed within DHBs (21.0%)(χ^2=10.6, p<0.001). However, nurses in hospital settings had higher attrition (50.0%) compared to those in community settings (37.0%). Nurses working part-time in 2006 had higher 5-year separation rates across all age bands compared to those working full-time (p<0.001). Attrition was highest among the following practice areas: nursing administration and management (58.0%), assessment and rehabilitation (58.0%), and continuing care (55.0%), compared to mental health (31.%), perioperative care (35.0%), and primary health care (38.0%).		
Price, 2016 [27]	Registered nurses employed in primary and public sectors; 2008/2011	138	Nurse attrition was measured through questions related to current employment status, country of employment, and intention to travel and work from 2008 to 2011.	In the fourth year of practice (2011), there was a statistically significant increase in the number of nurses employed in Australia (15.6%) compared to the baseline (7.4%)(χ^2=4.48, p=0.03). A significant	7.2%	Y

				association was found between the preferred area of work and NZ residence (p=0.04). Nurses without postgraduate qualifications had a higher attrition rate (29.0%) compared to those with higher qualifications.		
Reid, 2019 [33]	Physiotherapists ; 2019	1,207	Physiotherapist attrition was measured via questions relating to holding current registration requirements. Other instruments used included the Copenhagen Psychosocial Questionnaire, Effort-Reward Imbalance, and other customised questions.	The strongest correlates (r≥0.6) of attrition among physiotherapists include predictability and recognition; quality of leadership and recognition; quality of leadership and supervisor support, predictability and quality of leadership, recognition and supervisor support, role clarity and recognition, meaning or work and workplace commitment, CPD interference and family plans, CPD interference and ease of completing hours, APC cost and family plans, and work prospects and salary adequacy.	11.3%	Y
Shelker, 2014 [31]	Medical graduates (doctors) from Otago Faculty of Medicine and Auckland Medical Programme; 1999/2012	3,865	Tracking of graduate doctor registration through the NZ Medical Register from 1999 to 2011.	At **13 years, Health Sciences First-Year Students showed a 7.0% higher attrition rate than those in the Competitive Graduation Entry in Otago data (OR=1.36,**	25.9%	Y

				**p=0.04). Age at graduation was not significantly associated with attrition (p=0.31).** There was no significant difference in long-term attrition between Auckland and Otago data (p=0.48).		
Stokes, 2018 [43]	Occupational therapists; 2016/2017	1,059	Occupational therapists were asked survey questions related to their reasoning for leaving the profession.	The most common reasons occupational therapists leave the profession are parental leave (40.0%), followed by working conditions (38.0%), career change (31.01%), and going overseas (not quantified but common).	NR	N[b]
Wakelin, 2007 [30]	Lead maternity care midwives at one large urban region in NZ; 2006	94	Lead Maternity practice attrition was measured using a Likert scale and closed-ended questions.	The main reasons for leaving included exhaustion (66.0%), no time for self (41.0%), and medico-legal anxiety (28.0%).	25.5%	Y
[a]Sample size of measurement outcome assessed; [b]Excluded from meta-analyses due to no raw frequency data available; NR = Not Reported; χ^2=Chi squared statistic; r= Pearsons correlation coefficient; CPD = Continued Professional Development; NZ = New Zealand; DHB = District Health Board;						

Supplementary Table 3: Summary of studies examining intention-to-leave (n=26)

1st Author, Year	Profession and setting; recruitment year	Sample size[a]	Measurement instrument and methods	Significance of the factors related to intention-to-leave	Percentage reporting intention-to-leave	Inclusion in meta-analysis

ASMS, 2022 [52]	Senior doctors and dentists from public and private sectors; 2022	1,594	Intention-to-leave was measured using a future intentions question on a five point Likert scale. Components of satisfaction were also evaluated using a five point scale.	Significant predictors of turnover intention include dissatisfaction with the ability to choose one's work arrangement (22.9%, p=0.005), level of responsibility (23.0%, p=0.008) recognition (21.2%, p=0.016), physical working conditions (20.7%, p=0.025), and work hours (21.5%, p=0.031). Some satisfied respondents still intended to leave. Remuneration (p=0.23) and interactions with colleagues (p=0.86) were not significant predictors of turnover intention.	18.0% (within 5 years)	Y
Bradley, 2024[a] [41]	Early-career pharmacists from public and private sectors; 2019	416	The likelihood of staying in the pharmacy profession was measured on a five point Likert scale. Scores of 'very unlikely' and 'unlikely' to stay were used to calculate intention-to-leave.	NR	33.0% (within 5 years)	Y
Chambers, 2022 [36]	Psychiatrists working in public mental health services; 2021	342	A pre-validated question using a five point Likert scale was used to measure intention-to-leave. Scores of 'strongly agree' and 'agree' were used to	Psychiatrists experiencing high levels of burnout (p<0.001, OR=1.71) and job stress (p<0.001, OR=1.53) were significantly more likely to have a planned intention to leave.	23.0% (within 6-12 months)	Y

			calculate intention-to-leave prevalence. A single-item question from the Maslach Burnout Inventory was used to measure burnout, as well as a single-item measure of stress. Other variables, including job demands, support, and satisfaction, were measured using Likert scales.	Factors associated with higher intentions to leave include dissatisfaction with job resources ($p<0.001$, OR=5.03 for burnout), frequent covering of colleagues' caseloads ($p<0.001$, OR=2.11 for burnout), and poor resourcing ($p<0.001$, OR=0.49 for burnout).		
Clendon, 2011 [38]	Young nurses (aged 30 and under); 2010	674	Intent to leave was measured through a binary (yes or no) question. Personal and professional demographic and professional demographic variables were also assessed.	NR	10.3% (within 12 months)	Y
Domett, 2024 [55]	Doctors working in primary/community and hospital/secondary care; 2024	1,300	Turnover intention was measured using a multi-choice question where participants indicated the likelihood of doing each item. Other variables such as burnout, workload, support, and remuneration were also independently measured.	Hospital/secondary care doctors (30.0%) and community/primary care doctors (41.0%) had discussed leaving the profession with others. Planned reasons for leaving within the next 12 months include retirement (4.0%), moving to practice overseas (6.5%), and leaving the medical profession entirely (3.5%).	14.0% (within 12 months)	Y
Finlayson, 2007 [45]	Nurses working in publicly funded secondary	4,603	Intention to leave was measured through a single	Intention to leave was highly prevalent among younger nurses,	33.8% (within 12 months)	Y

	and tertiary hospitals; 2001		item within the Revised Nurse Work Index. Burnout was independently measured using the Maslach Burnout Inventory, alongside other items that assessed perceived changes in nursing structure.	with 56.6% of nurses aged 30 and under planning on leaving within the next year.		
Jiang, 2023 86]	Nurses; 2018/2019	294	Intention to leave was measured on a five point scale using the three item tool by Hanisch and Hulin.	Turnover intention was positively correlated with coworker incivility ($r=0.26$, $p<0.05$), supervisor incivility ($r=0.30$, $p<0.05$), doctor incivility ($r=0.26$, $p<0.05$), and patient/visitor incivility)r=0.18, p=0.05). Negative correlations were observed with the need for belongingness ($r=-0.34$, $p<0.05$), need for competence ($r=-0.09$, $p>0.05$), and need for autonomy $r=-0.55$, $p<0.05$). Mean of turnover prevalence=2.29.	NR	N[b]
Kalliath, 2001 [53]	Nurses belonging to surgical, medical, and acute wards of a large NZ hospital; 2001	250	Intention to leave was measured by a three item questionnaire developed by O'Driscoll and Beehr. Burnout was measured using two subscales from Maslach Burnout Inventory. Supervisor support was	Intention to quit was positively correlated with depersonalisation ($\beta=0.27$, $p<0.05$) and emotional exhaustion ($\beta=0.21$, $p<0.05$), and negatively correlated with supervisory support ($\beta=-0.30$, $p<0.01$). Planned departure within the next 6 months = mean 3.73.	NR	N[b]

			measured with a six item Likert scale version of the index of Organisational Reactions.	Planned departure within the next 12 months = mean 3.58.		
Lau, 2004 [37]	Vocationally registered psychiatrists working across metropolitan and non-metropolitan areas; 2001	159	The practice intentions of psychiatrists were measured through multi-choice questions and a three point Likert scale.	The top five reasons for prompting departure from NZ included higher remuneration (mean=1.35); better professional support and development (mean=1.26); improved career opportunities (mean=1.20); closer to family (mean=1.17); and stress/burnout (mean=1.19). Of those likely to practice outside of NZ, Australia was the most popular likely destination.	23.3% (within 10 years)	Y
Lynch, 2014 [34]	Medical laboratory professionals working within private and public anatomical pathology labs within the Auckland metropolitan area; 2014	66	Medical laboratory professionals were asked closed-ended questions regarding their intention-to-leave the profession.	Reasons for leaving included retirement (5.0% of the total sample) and career change (3.0% of the total sample).	8.0% (within 5 years)	Y
Moloney, 2018[a] [51]	Nurses; 2014/2015	2,876	Intention to leave was measured using a three item scale from Dotson, Dave, Cazier, and Spaulding. Various other measures relating to variables of interest were assessed using validated scales.	Intention to leave the profession was positively correlated with burnout (β=0.24, p<0.001), work-life interference (β=0.17, p<0.001), workload (β=0.13, p<0.001) and emotional demands (hindrances)(β=0.06, p<0.001).	NR	N[b]

				Negative correlations included engagement (β=-0.45, p<0.001), emotional demands (challenges) (β=-0.1, p<0.001, self-efficacy (β=-0.11, p<0.001), collegial support (β=-0.08, p<0.001), organisational support (β=-0.08, p<0.001), and autonomy (β=-0.06, p<0.001).		
Moloney, 2018[b] [50]	Nurses; 2014/2015	2,175	A binary (yes or no) question was used to determine nurses' intent to leave the profession as their financial situations improved. Factors associated with this intent were also identified.	Factors associated with intent to leave the profession included job satisfaction (β=-0.10, p<0.01), career orientation (β=-0.14, p<0.01), work engagement (β=-0.09, p<0.01), and burnout (β=0.09, p<0.01).	22.6% (when finances improve)	Y
Moloney, 2024 [54]	Nurses working in primary healthcare; 2019	231	Intention to leave the profession was measured by a three item scale from Dotson, Dave, Cazier, and Spaulding. Five other validated measures were also used to examine the following variables: thriving at work, self-efficacy, empowering leadership, perceived organisational support, and burnout.	Factors associated with the intent leave the profession include vitality (a component of thriving at work) (β=-0.29, p<0.05), empowering leadership (indirectly)(β=-0.07), and perceived organisational support (indirectly)(β=-0.08). Planned departure from the profession = mean 2.9	NR	N[b]
Ng, 1991 [87]	Nurses working in 19/20 hospital	1,002	Quitting intention was measured by	Quitting was correlated with marital status	16.2% (within 15 months)	Y

	health boards; 1988		two questions relating to the certainty and immediacy of quitting, scored on six and seven point scales, respectively. Scores from each item were summated to produce a final score.	(β=0.12, p<0.001), training (β=0.06, p<0.05), quitting intention (β =0.41, p<0.001), having an exjob (β=-0.06, p<0.05), ethnicity (β =0.04, p>0.05), housing mortgage (β =0.00, p>0.05), tenure (β =-0.01, p>0.05), and support facilities (β =-0.02, p>0.05).		
Pick, 2008 [88]	All ophthalmologists in NZ; 2007/2008	121	Ophthalmologists were asked about the timeframe of their retirement plans through a categorical Likert-style scale.	NR	23.1% (within 5 years)	Y
Price, 2016 [27]	Registered nurses employed in primary and public sectors; 2008/2011	138	Nurses' intention-to-leave was measured via questions relating to current employment status, country of employment, and intention to travel and work from 2008-2011.	There was a statistically significant increase in intention-to-leave in 2011 (37.0%) compared to the 2008 baseline (17.4%).	37.0% (within 12 months)	Y
Pharmaceutical Society NZ, 2024 [42]	Pharmacists employed in various pharmacy settings; 2024	431	Pharmacists' intention-to-leave was measured through a single-item categorical Likert-style scale.	Of community pharmacists, 51.0% have an intention-to-leave, compared to hospital pharmacists (40.0%), GP pharmacists (20.0%), and pharmacists working in non-patient-facing roles (29.0%).	49.2% (within 5 years)	Y
Psychology Workforce Task Group, 2017 [40]	Registered psychologists employed across various sectors; 2017	634	Psychologists rated their likelihood of leaving their current role on a five point	Top reasons for considering leading included: Better pay and working conditions, lack	40.0% (within 5 years)	Y

			Likert scale. Scores of ‘quite’ and ‘very’ likely were used to calculate overall turnover intention. Pharmacists who indicated any likelihood were asked to select up to four multi-choice reasons from a list of 25 common reasons for leaving a job.	of resources to do the job well, not feeling valued, distrust of management, poor communication with management, staff conflict, and lack of career advancement		
Ram, 2025 [48]	Internationally qualified nurses who completed a Competency Assessment Programme; 2023	323	Intention to leave was measured through two multi-choice items relating to intent to leave and reasons for this intent.	Reasons for leaving intention included family or personal reasons (n=131), better job opportunities (n=127), career advancement (n=45), attraction to a specific city or region (n=42), and familiarity with another healthcare system (n=16).	15.0% (unspecified timeframe)	Y
Ravenswood, 2021 [49]	Nurses working in care and support work; 2019	189	Intention to leave among nurses was measured using a Likert scale question. Reasons for this intent were captured through a multi-choice question. Scores of ‘agree’ and ‘strongly agree’ were used to calculate overall turnover intention. Nurses were asked to select one reason of 13 multi-choice options for	The most common reason for nurses intention-to-leave was stress/burnout (22.2%), followed by family reasons (13.3%), and employment conditions (12.8%).	33.8% (within 12 months)	Y

			leaving their job.			
Reid, 2019 [33]	Physiotherapists; 2019	1,207	The five item Turnover Intention Scale (TIS) was used to obtain intention-to-leave data. Item one scores of ‘usually’ or ‘always’ were summated to calculate overall intention-to-leave prevalence.	The strongest correlates ($r \geq 0.6$) of turnover intention among physiotherapists include poor leadership and lack of supervisor support, lack of recognition and role clarity, work life conflict, low satisfaction with personal needs fulfilment and weak interpersonal support and communication. Moderate correlates ($r=0.4$-0.6); inadequate work prospects and role fit, job dissatisfaction, weak workplace commitment, high work pace and emotional demands Weak correlates ($r<0.3$); CPD burden, job security	17.8% (unspecified timeframe)	Y
RNZCGP; 2023 [46]	General practitioners and rural hospital medicine doctors in NZ; 2022	3,281	Turnover intentions among GPs were measured through Likert scale questions. The intention-to-leave NZ and work was used to calculate overall turnover intention.	GPs aged 39 and younger are more likely to intend to leave NZ (16.0%) compared to older GPs aged over 65 (3.0%). Burnt-out GPs are also more likely to intend to **retire within 5 years** (40%) compared to the general GP population (37%) . Practice owners are more likely to retire within 5 years (46.0%) than employees (30.0%). Males are more likely to retire within 5	9.0% (within 5 years)	Y

				years (46.0%), vs females (30.0%)		
Taylor, 2020 [47]	Radiation therapists from all 10 public and private cancer centres in NZ; 2019	362	Intention to leave was measured via a composite of three binary (yes or no) questions. A preliminary analysis was conducted to identify key themes in workplace perception, which were used in conjunction with demographic variables within a logistic regression model.	Among those intending to leave. 31.0% will leave within the next 12 months, 25.0% within 1-2 years, 21.0% in 3-6 years, 4.0% after 7 years, and 19.0% when a role becomes available. Intention to leave the workplace was positively associated with workload satisfaction (OR=1.57, p<0.03), professional development (OR=2.39, p<0.01), and holding a bachelors' qualification (OR=0.41, p=0.04). Intention-to-leave the profession was negatively associated with older age (β=-0.02, p<0.01), higher workplace satisfaction (β=-0.20, p<0.01), and having pride in being a radiation therapist (β=-0.12, p<0.01). Positive correlations were found with variety of work (β=0.14, p<0.01), and years of experience (β=0.02, p<0.01).	33.0% (varied timeframes)	Y
Wakelin, 2007 [30]	Lead maternity care midwives at one large urban region in NZ; 2006	70	Intention to leave Lead Maternity Practice was measured using Likert scale and closed ended questions.	Reasons for considering leaving include exhaustion (30.0%), no time for self (17.0%), and medico-legal anxiety (11.0%).	54.3% (within 5 years)	Y

Walker, 2016 [89]	Asian nurses from various healthcare sectors; 2015	562	Turnover intention among Asian nurses was measured through closed-ended, multi-choice questions (yes, no, or unsure). Demographic questions were also included in the survey.	NR	12.0% (unspecified timeframe)	Y
Walker, 2017 [90]	Members of the NZ Nurses Organisation; 2016	628	Nurses' intention-to-leave the workforce was measured through dichotomous (yes or no) questions. Retirement intentions were used to calculate the total prevalence of turnover intention. Other factors, including burnout, workload, and career satisfaction, were measured using Likert scales.	NR	16.7% (within 5 years)	Y

[a]Sample size of measurement outcome assessed; [b]Excluded from meta-analyses due to no raw frequency data available; NR = Not Reported; OR = Odds Ratio;
r= Pearsons correlation coefficient; β= standardised regression coefficient; CPD = Continued Professional Development; NZ = New Zealand; ASMS = Association of Salaried Medical Specialists; NZWIM = New Zealand Women in Medicine; RNZCP = Royal New Zealand College of General Practitioners; NZNO = New Zealand Nurses Organisation
